# Acknowledgement to Reviewers of *Biology* in 2013

**DOI:** 10.3390/biology3010176

**Published:** 2014-02-27

**Authors:** Biology Editorial Office

**Affiliations:** MDPI AG, Klybeckstrasse 64, CH-4057 Basel, Switzerland

The editors of *Biology* would like to express their sincere gratitude to the following reviewers for assessing manuscripts in 2013:

Vecoli, Cecilia
Moore, Adrian W.
Sachidanandam, Ravi
Ciaudo, Constance
Abi-Gerges, Najah
Niclou, Simone P.
Zhang, Zhenwei
Price, Steve
Schmeller, Dirk S.
Werner, Thomas
Diaz, Pilar
Cho, William
Sundram, Vasudha
Margesin, Rosa
Fritsen, Christian H.
Walker, Virginia
Moeseneder, Markus
Witte, Ursula
Tutino, Luisa M.
Männistö, Minna K.
Edwards, Kate A.
Favero-Longo, Sergio e.
Gardali, T.
Hart, Kris M.
De Los Ríos, Asunción
Weiss, Günter
Blackwell, Jenefer M.
Gazouli, M.
De Vendittis, Emmanuele
Schmitz, Jürgen
Edwards, Scott
Foreman, Christine M.
Morgan-Kiss, Rm
Chemin, I.
Ray, Ranjit
Hole, David
Møller, Anders Pape
Ellwood, Elizabeth R.
Deslippe, Julie R.
Powell, S. M.
Weimer, Paul J.
Hinz, Boris
Tomasek, James J.
Leask, Andrew
Gouin, Sebastien
Bouckaert, Julie
Ofek, Itzhak
Thomas, David
Underwood, Graham J. C.
Sintes, Eva
Levin, Michael
Tani, Kenzaburo
Szalay, Aladar A.
Hartkopf, Andreas D.
Knowles, Emily J.
Boletzky, Sigurd
Barord, Gregory
Selbmann, Laura
Klymkowsky, Michael
Sime-Ngando, Télesphore
Maček, Irena
Barbieri, Elena
Yutaka, Kawarabayasi
Papaleo, Elena
Schoch, Conrad L.
De Hoog, Sybren
Nelsen, M. P.
Gazis, Romina
Leon Sicarios, Nidia
Farfan, Mauricio J.
Rascón-Cruz, Quintín
Pieters, Roland J.
O'callaghan, John
Voulhoux, Rome
Remaut, Han
Turner, David P. J.
Pelicic, Vladimir
Barocchi, Michele A.
Zamarin, Dmitriy
Takamatsu, Daisuke
Samal, Siba K.
Griffiths, Charles
Dundr, Miroslav
Elliott, David
Pieczynski, Wojciech
Kramer, Axel
Radke, C. J.
Bazaka, Katia
Mimuro, Hitomi
Fröhlich, Holger
Braun, Edward L.
Rastelli, Eugenio
Price, Buford
Stibal, Marek
Kim, Hak Jun
Li, Meng
Giovannelli, Donato
Kalali, Behnam
Habib, Tanwir
Studholme, David
Choi, Hyung Won
Prade, Rolf A.
Lohse, Marc
Sese, Jun
Thomas, Wendy E.
Ugorski, Maciej
Sokurenko, Evgeni V.
Barišić, Lidija
Bošnački, Dragan
Häder, Donat-P.
Rautio, Milla
Oelschlaeger, Tobias
Konkel, Michael E.
Zhu, Jing
Shannon, Paul
Pico, Alex
Mucherino, Antonio
Shanks, Robert M. Q.
Omali, Negar Babaei
Zautner, Andreas
de Folter, Stefan
Vandenbussche, Michiel
Heimer, Susan
Wichmann, Søren
Ritchie, Dave
Shatsky, Maxim
Jagodzinski, Filip
Feizi, Soheil
Liu, Rong
Poland, Jesse
Wang, Xiaowu
Cremer, Thomas
Swiezewski, Szymon
Chevalier, Christian
Harrington, Timothy
Felix, Joel
Akhunov, Eduard
Raynaud, Cécile
De Veylder, Lieven
Gossett, Dalton
Mellon, Jay
Lunter, Gerton
Guevel, Laetitia
Hathout, Yetrib
Marcus, Katrin
Fan, Chuanzhu
Fares, Mario A.
Morgan-Kiss, Rachel
Job, Dominique
Mueller, Kerstin
Yano, Ryoichi
Kroger, R.
Gissi, Carmela
Santos, Ana Paula
Luo, Chongyuan
Guyot, Romain
Behrens, Sarah
Gestwicki, Jason E.
Srivastava, Vibha
Rampelotto, Pabulo Henrique
Malmström, Lars
Bregitzer, Phil
Gambino, G.
Wu, Meng
Lam, Ying Wai
Henrich, Curtis J.
Knip, Danielle M.
Foret, Frantisek
Nash, Michael A.

